# Fine-Tuning of the Grain Size by Alternative Splicing of *GS3* in Rice

**DOI:** 10.1186/s12284-022-00549-5

**Published:** 2022-01-11

**Authors:** Lei Liu, Ying Zhou, Feng Mao, Yujuan Gu, Ziwei Tang, Yi Xin, Fuxia Liu, Tang Tang, Hui Gao, Xiangxiang Zhao

**Affiliations:** 1 Jiangsu Key Laboratory for Eco-Agriculture Biotechnology Around Hongze Lake, Huaiyin Normal University, Huai’an, 223300 China; 2 Jiangsu Collaborative Innovation Center of Regional Modern Agriculture and Environment Protection, Huaiyin Normal University, Huai’an, 223300 China; 3 College of Marine Resources and Environment, Hebei Normal University of Science and Technology, Qinhuangdao, 066600 China

**Keywords:** Grain size, *GS3*, Alternative splicing, Gene expression, Post-transcriptional level

## Abstract

Grain size is subtly regulated by multiple signaling pathways in rice. Alternative splicing is a general mechanism that regulates gene expression at the post-transcriptional level. However, to our knowledge, the molecular mechanism underlying grain size regulation by alternative splicing is largely unknown. GS3, the first identified QTL for grain size in rice, is regulated at the transcriptional and post-translational level. In this study, we identified that *GS3* is subject to alternative splicing. *GS3.1* and *GS3.2*, two dominant isoforms, accounts for about 50% and 40% of total transcripts, respectively. *GS3.1* encodes the full-length protein, while *GS3.2* generated a truncated proteins only containing OSR domain due to a 14 bp intronic sequence retention. Genetic analysis revealed that GS3.1 overexpressors decreased grain size, but GS3.2 showed no significant effect on grain size. Furthermore, we demonstrated that GS3.2 disrupts GS3.1 signaling by competitive occupation of RGB1. Therefore, we draw a conclusion that the alternative splicing of *GS3* decreases the amount of *GS3.1* and GS3.2 disrupts the GS3.1 signaling to inhibit the negative effects of GS3.1 to fine-tune grain size. Moreover, the mechanism is conserved in cereals rather than in Cruciferae, which is associated with its effects on grain size. The results provide a novel, conserved and important mechanism underlying grain size regulation at the post-transcriptional level in cereals.

**Supplementary Information:**

The online version contains supplementary material available at 10.1186/s12284-022-00549-5.

## Findings

Alternative splicing (AS) is a general mechanism that regulates gene expression at the post-transcriptional level in eukaryotes. AS produces multiple mRNA variants from a single locus by selective usage of different splice sites (Sanchez et al. 2011). AS variants are usually translated into truncated proteins to play antagonistic roles or degraded by the nonsense-mediated decay (NMD) pathway to negatively control the amount of normal proteins, which expands the transcriptome and/or proteome diversities to fine-tune cellular processes (Liu et al. 2021).

G-protein signaling regulates grain size in plants. In rice, there are one Gα (RGA1), one Gβ (RGB1), and five Gγ (RGG1, RGG2, GS3, DEP1 and GGC2) (Xu et al. 2019). Among these components, GS3 is the only one that is unquestionable to negatively regulates grain size (Sun et al. 2018). Further analysis demonstrated that the C-terminus of GS3 shows an inhibitory effect on the function of the N-terminal domain with regard to grain size (Mao et al. 2010), which can be explained by the C-terminal tail-mediated endosomal degradation via E3 ligase CLG1 (Sun et al. 2018; Yang et al. 2021).

**Fig. 1:**
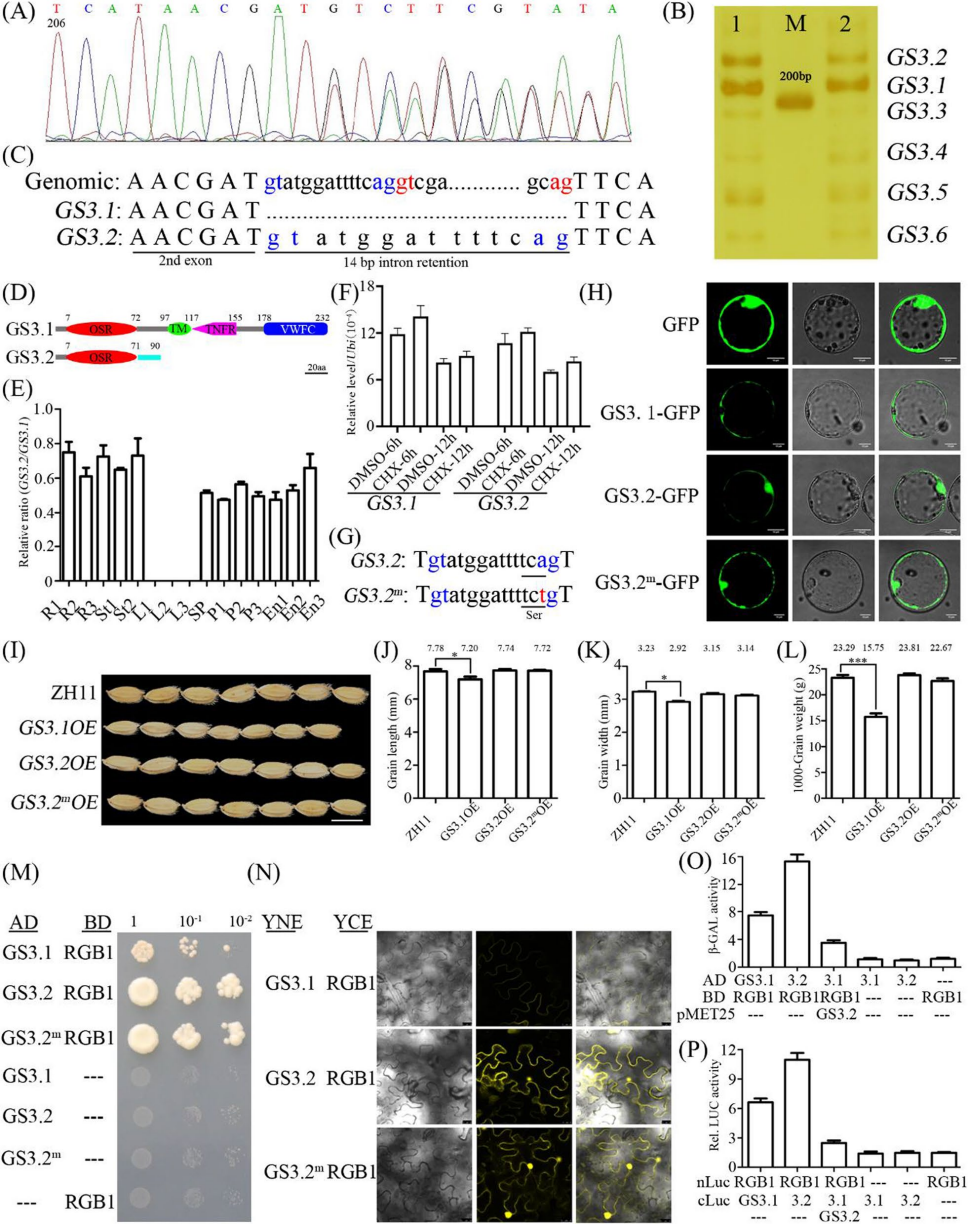
Alternative splicing of *GS3* fine-tunes grain size in rice. **A** Sequencing of PCR products of *GS3*. **B** *GS3* AS variants are shown by PAGE. Lane 1 and 2 show 40 and 35 cycles, respectively. M, DNA ladder. **C** Sequence comparison of *GS3.1* and *GS3.2*. **D** Protein structures of GS3.1 and GS3.2. **E** Ratio of *GS3.2*/*GS3.1* in different tissues. **F** *GS3.2* expression level under CHX treatment. **G** Construct of GS3.2^m^ by A-T substitution in 3’ splice site. **H** Subcellular localization of GS3.1, GS3.2 and GS3.2^m^ in rice protoplasts. **I** Phenotypic comparison of grain length among *GS3.1* and *GS3.2* overexpressors. Bar = 1 cm. **J** Grain length, **K** grain width and **L** 1000-grain weight of the genotypes tested. Data are given as mean ± SEM. *p < 0.01; ***p < 0.001. **M** Interactions between GS3 AS variants and RGB1 tested by yeast-two-hybrid and **N** by BiFC. **O** GS3.2 disrupts the interaction between GS3.1 and RGB1 tested by yeast-three-hybrid and **P** by luciferase activity assay

Besides the transcriptional and post-translational regulation, *GS3* undergoes AS (Fig. 1A and Additional file 1: Fig. S1). The visible bands were sequenced and confirmed to be *GS3* AS variants, namely *GS3.1*-*GS3.6*, respectively (Fig. 1B). The AS types occurred in *GS3* include intron retention, exon skipping and alternative 3' splice site (Additional file 1: Fig. S2). Moreover, all the selective splice sites were consensus with 5'GT-3'AG sequences (Additional file 1: Fig. S2). In line with the observation in gel electrophoresis (Fig. 1B), clone number analysis indicated *GS3.1* and *GS3.2* absolutely dominate among the variants (Additional file 1: Fig. S3). Further analysis revealed that the AS mechanism is conserved in cereals but not in Cruciferae (Additional file 1: Table S1), which may be explained by the opposite effects of *GS3* homologs on grain size in cereals and Cruciferae (Li et al. 2012).

*GS3.2*, the dominant AS variants that introduced a 14 bp intron to lead to a premature stop codon by frameshift (Fig. 1C), putatively encodes a truncated protein only containing the OSR domain (Fig. 1D), which is not found in natural allelic variation of GS3. Expression analysis revealed that *GS3.2* share the same patterns with *GS3.1*, albeit with lower expression level (Fig. 1E and Additional file 1: Fig. S4). Since translation is required for NMD, we analyzed *GS3.2* transcript from plants grown in the presence of cycloheximide, an inhibitor of translation (Sureshkumar et al. 2016). No significant enrichment of *GS3.2* transcripts were observed, suggesting that GS3.2 are not targeted for degradation by the NMD pathway (Fig. 1F). To confirm the result, we next performed transient expression and analyzed its subcellular localization. To avoid the splicing of the retained 14 bp sequences, a construct with a A-T substitution in the 3' splice site, namely GS3.2^m^, was generated (Fig. 1G). Notably, compared with the plasma localization of GS3.1, both GS3.2 and GS3.2^m^ were localized in the plasma membrane and nuclear (Fig. 1H), indicating that the function of GS3.2 may be distinguished from that of GS3.1.

To uncover the biological significance of AS occurred in *GS3*, *GS3.1*, *GS3.2* and GS3.2^m^ driven by *Actin* promoter were introduced into Zhonghua 11 (ZH11). *GS3.1*, *GS3.2* and *GS3.2*^*m*^ overexpressors were identified (Additional file 1: Fig. S5). Compared with ZH11, overexpression of *GS3.1* resulted in small grains, but GS3.2 and GS3.2^m^ overexpressors showed no significant change (Fig. 1I). Further statistical analysis confirmed that elevated *GS3.1* reduces grain length by 8.0%, grain width by 10.6%, and 1000-grain weight by 47.9%, but elevated GS3.2 and GS3.2^m^ show no obvious difference (Fig. 1J–L and Additional file 1: Table S2), indicating the antagonistic roles for GS3.1 and GS3.2 in grain size regulation. It is well studied that GS3 associates with RGB1 to negatively regulate grain size (Sun et al. 2018). The question whether GS3.2 associates with RGB1 is raised. First, yeast two-hybrid assay was performed to detect the interaction between GS3.2 and RGB1. The result showed that the interaction between GS3.2 and RGB1 is much stronger than that between GS3.1 and RGB1 (Fig. 1M), which is indicated by the growth strength in the dropout medium. Next, BiFC assay confirmed the direct interaction between GS3.2 and RGB1 (Fig. 1N). According to the above results, it is reasonable to speculate that GS3.2 attenuates GS3.1 activity by competitively interacting with RGB1. To test the hypothesis, yeast three-hybrid assay was conducted. Obviously, the interaction between GS3.1 and RGB1 is subdued by GS3.2 (Fig. 1O). In addition, such competition was also observed in luciferase activity assay using rice protoplast (Fig. 1P), indicating GS3.2 disrupts GS3.1 function via occupation of RGB1.

In summary, our results revealed that GS3 is alternatively spliced and the AS mechanism is evolutionarily conserved in cereals but not in Cruciferae. *GS3.1* negatively regulates grain size (Fig. 1I–N), while the majority AS variants *GS3.2*, which accounts for about 40% of *GS3* transcript, displays no negative effects on grain size (Fig. 1I–N). Moreover, GS3.2 attenuates GS3.1 activity by competitively interacting with RGB1 (Fig. 1O, P). Collectively, it is reasonable to conclude that the alternative splicing of *GS3* decreases the amount of *GS3.1* and GS3.2 disrupts the GS3.1 signaling to inhibit the negative effects of GS3.1 to fine-tune grain size.

Grain size is regulated by multiple signaling pathways mainly at the transcriptional and post-translational level (Li et al. 2019; Zuo and Li 2014). This study shows grain size is fine-tuned by AS of *OsGS3* at the post-transcriptional level. The results provide a novel, conserved and important mechanism underlying grain size regulation in cereals.

## Supplementary Information

**Additional file 1: Fig. S1.** *GS3* is subject to alternative splicing. (A) *GS3* AS variants were shown by agarose gel electrophoresis. (B) *GS3* AS variants were sequenced by reverse primer. (C) *GS3* AS variants from Huaidao 5 were sequenced by the forward primer. **Fig. S2** Structures and sequences of *GS3* alternative splicing variants. (A) Structures of *GS3* alternative splicing variants. Gray boxes represent UTR. Black and red boxes indicate exons. Gray and blue lines denote introns. (B) Sequences of *GS3* alternative splicing variants. **Fig. S3**. Clone number of *GS3* alternative splicing variants. **Fig. S4**. Expression pattern analysis of (A) *GS3.1* and (B) *GS3.2* by qRT-PCR. R1-3, root in seedling, tillering and heading stage, respectively. St1-2, stem in elongation and heading stage, respectively. L1-3, leaf in seedling, tillering and heading stage, respectively. Sp, spikelet. P1-3, panicles with 2 mm, 3 cm and 5 cm length, respectively. En1-3, Endosperm of 3, 12 and 20 days after pollination, respectively. The results from three biological replicates are consistent. Data are shown as mean ± SEM from three technical replicates. **Fig. S5**. Expression analysis of *GS3.1* and *GS3.2*^*m*^ overexpressors. (A) Expression level analysis of *GS3.1* and *GS3.2*^*m*^ overexpressors by qRT-PCR. Ubiquitin was used as internal control. Data are shown as mean ± SEM. (B) Sequencing of the amplification products from *GS3.2 *and *GS3.2.*^*m*^ The red box indicated the mutation between *GS3.2* and *GS3.2*^*m*^. **Table S1**. Information of alternative splicing of *GS3* homologs and effects on grain size. **Table S2**. Summary of grain traits of *GS3* variants overexpressors. **Table S3**. List of primers used in this study.

## Data Availability

The data sets supporting the results of this article are included within the article and its additional files.

## Abbreviations

AS: Alternative splicing
NMD: Nonsense-mediated decay
RGA1: Rice G protein alpha subunit 1
RGB1: Rice G protein beta subunit 1
RGG1: Rice G protein gamma 1
RGG2: Rice G protein gamma 1
GS3: Grain size 3
DEP1: Dense and erect panicle 1
CLG1: Chang Li Geng 1
