# Supplementary material for: Fine-Tuning of the Grain Size by Alternative Splicing of GS3 in Rice
Source: Rice (N Y). 2022 Jan 11;15:4. doi: 10.1186/s12284-022-00549-5 (PMC8752655; doi:10.1186/s12284-022-00549-5)
Supplement: Supplementary file 1 — Additional file 1: Fig. S1. GS3 is subject to alternative splicing. (A) GS3 AS variants were shown by agarose gel electrophoresis. (B) GS3 AS variants were sequenced by reverse primer. (C) GS3 AS variants from Huaidao 5 were sequenced by the forward primer. Fig. S2 Structures and sequences of GS3 alternative splicing variants. (A) Structures of GS3 alternative splicing variants. Gray boxes represent UTR. Black and red boxes indicate exons. Gray and blue lines denote introns. (B) Sequences of GS3 alternative splicing variants. Fig. S3. Clone number of GS3 alternative splicing variants. Fig. S4. Expression pattern analysis of (A) GS3.1 and (B) GS3.2 by qRT-PCR. R1-3, root in seedling, tillering and heading stage, respectively. St1-2, stem in elongation and heading stage, respectively. L1-3, leaf in seedling, tillering and heading stage, respectively. Sp, spikelet. P1-3, panicles with 2 mm, 3 cm and 5 cm length, respectively. En1-3, Endosperm of 3, 12 and 20 days after pollination, respectively. The results from three biological replicates are consistent. Data are shown as mean ± SEM from three technical replicates. Fig. S5. Expression analysis of GS3.1 and GS3.2m overexpressors. (A) Expression level analysis of GS3.1 and GS3.2m overexpressors by qRT-PCR. Ubiquitin was used as internal control. Data are shown as mean ± SEM. (B) Sequencing of the amplification products from GS3.2 and GS3.2.m The red box indicated the mutation between GS3.2 and GS3.2m. Table S1. Information of alternative splicing of GS3 homologs and effects on grain size. Table S2. Summary of grain traits of GS3 variants overexpressors. Table S3. List of primers used in this study. [file 12284_2022_549_MOESM1_ESM.docx]

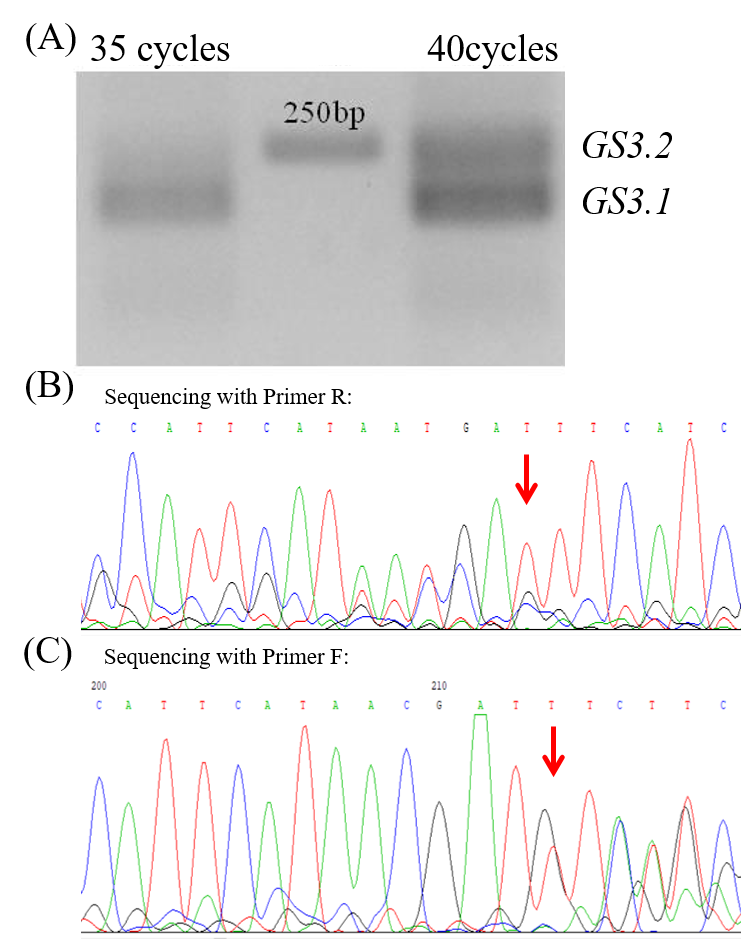


Supplemental Figure 1 *GS3* is subjective to alternative splicing.

(A) *GS3* AS variants were shown by agarose gel electrophoresis.

(B) *GS3* AS variants from ZH11 were sequenced by the reverse primer.

(C) *GS3* AS variants from Huaidao 5 were sequenced by the forward primer.


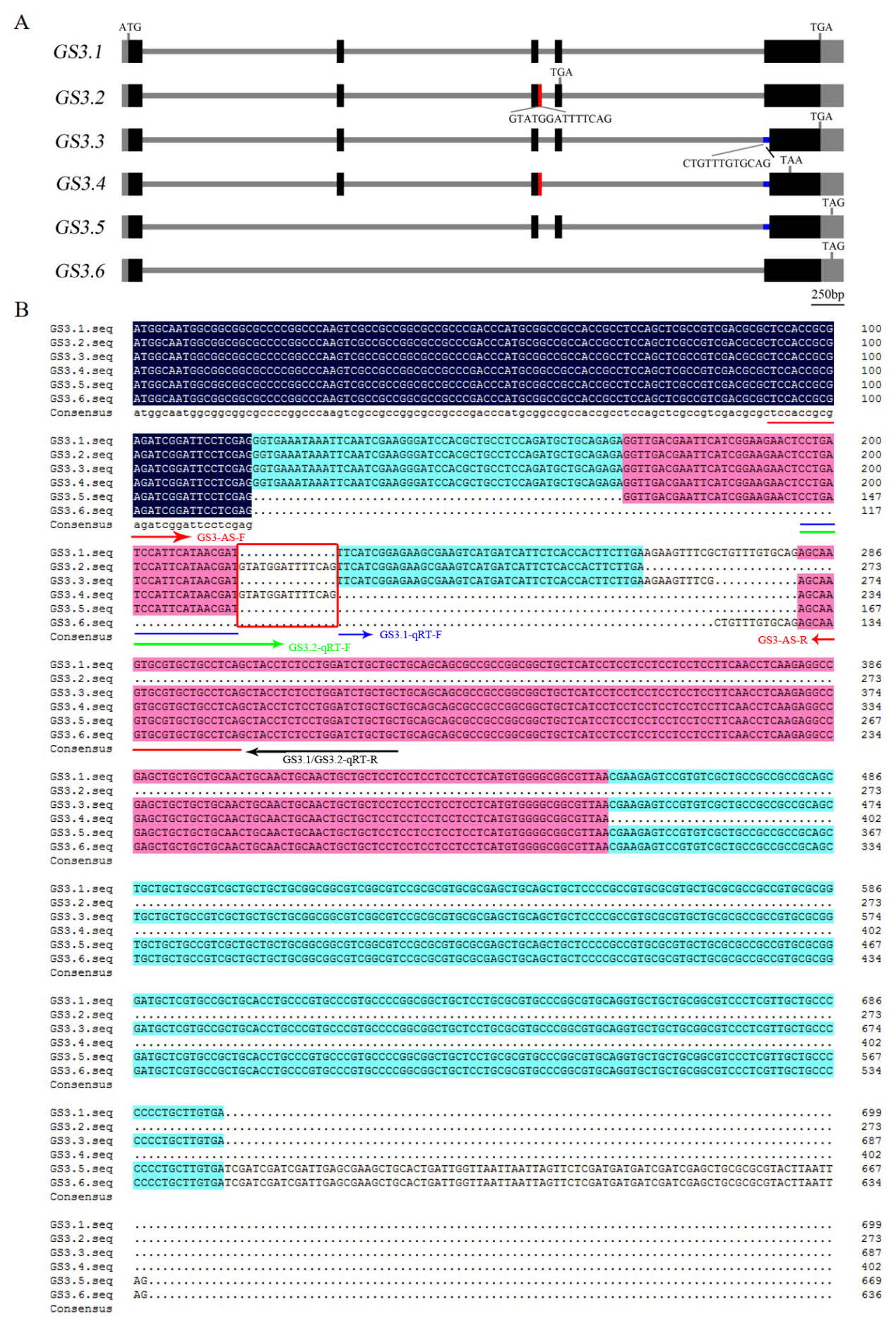


Supplemental Figure 2 Structures and sequences of *GS3* alternative splicing variants.

(A) Structures of *GS3* alternative splicing variants. Gray boxes represent UTR. Black and red boxes indicate exons. Gray and blue lines denote introns.

(B) Sequences of *GS3* alternative splicing variants. The red arrows indicate the primers for the amplification of *GS3* alternative splicing isoforms. The blue arrow (forward) and the black arrow (reverse) indicate the primers for *GS3.1* amplification. The green arrow (forward) and the black arrow (reverse) indicate the primers for *GS3.2* amplification. The red box indicates the retained intron sequences (14 bp) in *GS3.2*.


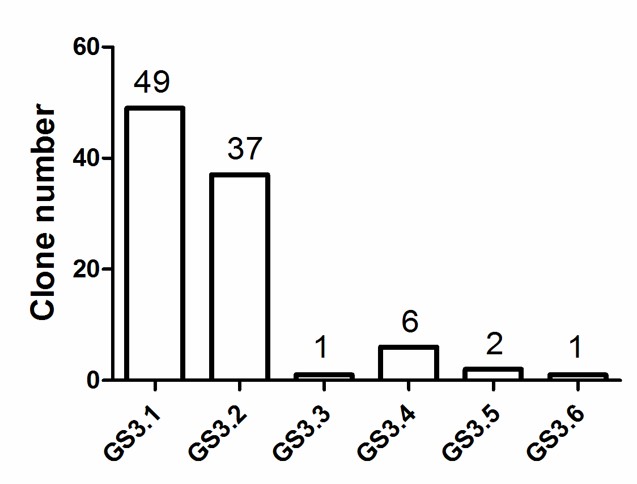


Supplemental Figure 3 Clone number of *GS3* alternative splicing variants.


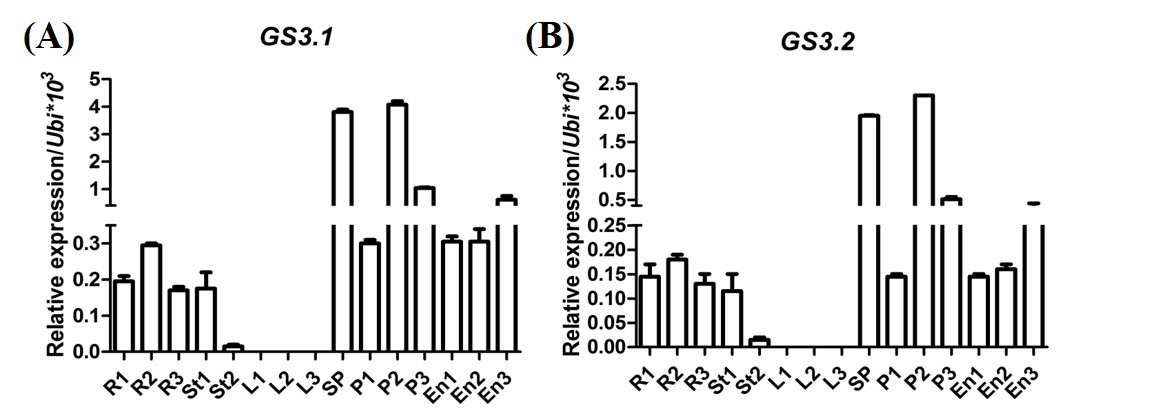


Supplementary Figure 4 Expression pattern analysis of (A) GS3.1 and (B) GS3.2 by qRT-PCR.

R1-3, root in seedling, tillering and heading stage, respectively.

St1-2, stem in elongation and heading stage, respectively.

L1-3, leaf in seedling, tillering and heading stage, respectively.

Sp, spikelet. P1-3, panicles with 2 mm, 3 cm and 5 cm length, respectively.

En1-3, Endosperm of 3, 12 and 20 days after pollination, respectively.

The results from three biological replicates are consistent.

Data are shown as mean ± SEM from three technical replicates.


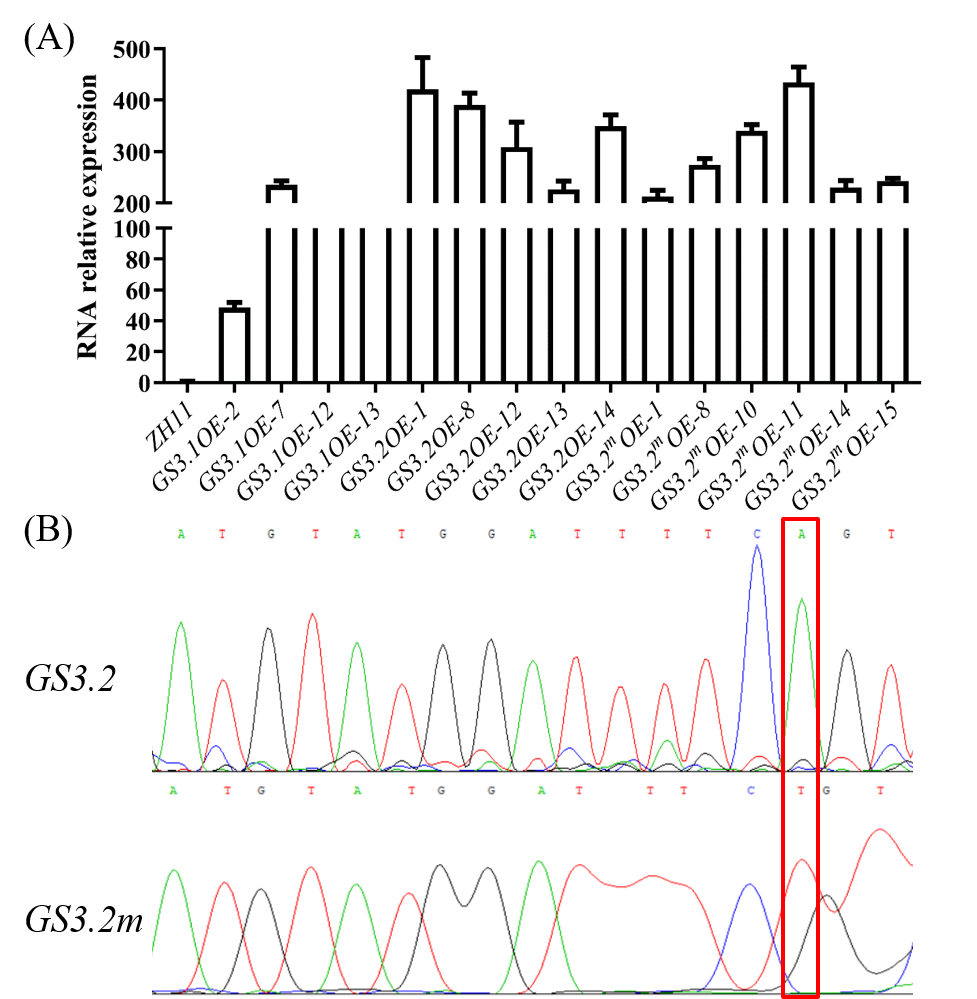


Supplemental Figure 5 Expression analysis of *GS3.1* and *GS3.2(m)* overexpressors.

(A) Expression level analysis of *GS3.1* and *GS3.2(m)* overexpressors by qRT-PCR.

Ubiquitin was used as internal control. Data are shown as mean ± SEM.

(B) Sequencing of the amplification products from *GS3.2* and *GS3.2m*. The red box indicated the mutation between *GS3.2* and *GS3.2m*.

Supplemental Table 1 Summary of grain traits of *GS3* variants overexpressors.

| Genotype | Grain length (mm) | Grain width (mm) | 1000-Grain weight (g) |
| --- | --- | --- | --- |
| ZH11-1 | 7.82 | 3.25 | 22.75 |
| ZH11-2 | 7.75 | 3.22 | 23.83 |
| *GS3.1OE-2* | 7.29 | 2.84 | 16.48 |
| *GS3.1OE-7* | 6.72 | 2.91 | 13.79 |
| *GS3.1OE-12* | 7.47 | 2.99 | 16.18 |
| *GS3.1OE-13* | 7.32 | 2.94 | 16.54 |
| *GS3.2OE-1* | 8.01 | 3.18 | 24.73 |
| *GS3.2OE-8* | 7.59 | 3.10 | 23.27 |
| *GS3.2OE-12* | 7.51 | 3.03 | 24.08 |
| *GS3.2OE-13* | 7.74 | 3.21 | 22.89 |
| *GS3.2OE-14* | 7.84 | 3.25 | 24.06 |
| *GS3.2mOE-1* | 7.61 | 3.05 | 21.58 |
| *GS3.2mOE-8* | 7.89 | 3.12 | 23.34 |
| *GS3.2mOE-10* | 7.78 | 3.13 | 22.34 |
| *GS3.2mOE-11* | 7.88 | 3.23 | 24.61 |
| *GS3.2mOE-14* | 7.63 | 2.98 | 21.09 |
| *GS3.2mOE-15* | 7.72 | 3.16 | 23.06 |

Supplemental Table 2 Information of alternative splicing of *GS3* homologs and effects on grain size.

| Species | Effects on grain size | Reference | Alternative splicing |
| --- | --- | --- | --- |
| *Arabidopsis thaliana* L. | Positive | Li et al., 2012 | No |
| *Brassica napus* L. | Positive | Li et al., 2012 | No |
| *Oryza sativa* L. | Negative | Sun et al., 2018 | Yes |
| *Triticum aestivum* L. | Negative | Zhang et al., 2020 | Yes |
| *Hordeum vulgare* L. | ND |  | Yes |
| *Brachypodium distachyon* L. | ND |  | Yes |
| *Oryza rufipogen* | ND |  | Yes |
| *Triticum urartu* | ND |  | Yes |
| *Aegilops speltoides* | ND |  | Yes |
| *Aegilops tauschii* | ND |  | Yes |

| Primer name | Primer sequence (presented 5’ to 3’) | Purpose |
| --- | --- | --- |
| GS3-seq-F | CTCCATTATCGGAACTTCGG | Amplification of GS3 for sequencing |
| GS3-seq-R | AACCAATCAGTGCAGCTTCG |  |
| GS3-AS-F | CTCCACCGCGAGATCGGA | AS of GS3 for electrophoresis |
| GS3-AS-R | CTGAGGCAGCACGCACTTG |  |
| GS3.1-qRT-F | CCTGATCCATTCATAACGATTTCA | Specific amplification of *GS3.1* expression |
| GS3.1-qRT-R | GCAGCAGATCCAGGAGAGGTAG |  |
| GS3.2-qRT-F | CCTGATCCATTCATAACGATGTATG | Specific amplification of *GS3.2* expression |
| GS3.2-qRT-R | GCAGCAGATCCAGGAGAGGTAG |  |
| GS3.1-1390F | GGGGTACCATGGCAATGGCGGCGGCGCC | Construct for *GS3.1* transformation |
| GS3.1-1390R | GGACTAGTCAAGCAGGGGGGGCAGC |  |
| GS3.2-1390F | GGGGTACCATGGCAATGGCGGCGGCGCC | Construct for *GS3.2* transformation |
| GS3.2-1390R | GGACTAGTAGAAGTGGTGAGAATGATCAT |  |
| AD-GS3.1-F | GGAATTCCATATGATGGCAATGGCGGCGGCGCC | Yeast two hybrid |
| AD-GS3.1-R | CGAGCTCTCACAAGCAGGGGGGGCAGC |  |
| AD-GS3.2-F | GGAATTCCATATGATGGCAATGGCGGCGGCGCC | Yeast two hybrid |
| AD-GS3.2-R | CGAGCTCTCAAGAAGTGGTGAGAATGATCAT |  |
| pAD-GS3.1-F | TCCCCCGGGGATGGCAATGGCGGCGGCGCC | Yeast three hybrid |
| pAD-GS3.1-R | TCCCCCGGGTCACAAGCAGGGGGGGCAGC |  |
| pAD-GS3.2-F | GAAGATCTATGGCAATGGCGGCGGCGCC | Yeast three hybrid |
| pAD-GS3.2-R | GAAGATCTTCAAGAAGTGGTGAGAATGATCAT |  |
| GS3.1-LUCF | GCTCTAGAATGGCAATGGCGGCGGCGCC | *LUC* assay |
| GS3.1-LUCR | GAAGATCTCAAGCAGGGGGGGCAGC |  |
| GS3.2-LUCF | GCTCTAGAATGGCAATGGCGGCGGCGCC | *LUC* assay |
| GS3.2-LUCR | GAAGATCTAGAAGTGGTGAGAATGATCAT |  |

Supplemental Table 3 List of primers used in this study.
